# Factors associated with inadequate iron supplementation among pregnant women in the Brazilian Unified Health System: a cross-sectional study

**DOI:** 10.1186/s12884-026-09437-y

**Published:** 2026-07-01

**Authors:** Isadora Parizotto Dimas de Souza, Daniela Bertotti, Catarina Parizotto Dimas de Souza, Dante Chiaradia Maule, Lilian de Paiva Rodrigues Hsu

**Affiliations:** Faculdade de Ciências Médicas da Santa Casa de São Paulo, São Paulo, Brazil

**Keywords:** Gestational anemia, Iron supplementation, Prenatal care, Public health

## Abstract

**Background:**

Gestational anemia remains an important public health problem, especially in developing countries, and is associated with adverse maternal and perinatal outcomes. Prophylactic iron supplementation during pregnancy is a simple and effective strategy to prevent this condition and is routinely recommended during prenatal care. In Brazil, supplements are provided free of charge through the public health system; however, adherence to treatment remains variable. This study aimed to identify the sociodemographic and healthcare-related factors associated with inadequate iron supplementation among pregnant women receiving care in the public health system.

**Methods:**

This cross-sectional study was conducted between March 2023 and May 2024 at the Maternity Unit of the Irmandade da Santa Casa de Misericórdia de São Paulo, a quaternary teaching hospital located in São Paulo, Brazil, and a reference center for obstetric care within the Brazilian Unified Health System (SUS). Women aged ≥ 18 years who were evaluated within 24 h after delivery were eligible. The exclusion criteria included fetal death, multiple pregnancies, and the presence of major fetal malformations. Data, including sociodemographic information, clinical history, prenatal care characteristics, and iron supplementation during pregnancy, were obtained through a structured questionnaire administered during the immediate postpartum period.

**Results:**

A total of 381 patients were included. Overall, 176 women (46.2%) had adequate iron supplementation during pregnancy and 205 (53.8%) had inadequate supplementation. Among the variables analyzed, drug use during pregnancy was the only sociodemographic factor that was significantly associated with inadequate iron supplementation (*p* = 0.001 crude OR 2.93, 95% CI 1.57–5.48). A trend toward greater adherence was observed among pregnant women followed exclusively within the public health system than among those receiving private, mixed, or overseas prenatal care, although this difference was not statistically significant. In multivariable logistic regression, self-reported drug use (aOR 2.54, 95% CI 1.33–4.87; *p* = 0.005), non-use of other vitamins during pregnancy (aOR 6.00, 95% CI 2.45–14.72; *p* < 0.001) and inadequate prenatal care (aOR 0.49 for adequate vs. inadequate prenatal care, 95% CI 0.25–0.94; *p* = 0.033) remained independently associated with inadequate iron supplementation.

**Conclusions:**

Inadequate iron supplementation during pregnancy is associated mainly with social and behavioral vulnerability, particularly drug use. These findings suggest that adherence depends not only on the availability of supplements but also on the quality of prenatal care and professional guidance. Public health strategies aimed at health education, strengthening primary care, and longitudinal follow-up of pregnant women may contribute to improving adherence and reducing the prevalence of gestational anemia.

**Supplementary Information:**

The online version contains supplementary material available at https://doi.org/10.1186/s12884-026-09437-y.

## Background

One of the goals established for 2030by the United Nations regarding global health and well-being is the reduction of maternal and perinatal mortality [1]. In this context, the presence of anemia during pregnancy represents an important indicator of maternal morbidity and mortality, affecting more than one-third of pregnant women in developing countries [2]. However, in most cases, this condition can be substantially prevented through iron supplementation, which is a relatively simple intervention in prenatal care [3]. Therefore, analyzing the epidemiological profile of women who do not adequately adhere to iron supplementation during pregnancy is a relevant topic for aligning public policies and healthcare practices within the Brazilian Unified Health System (Sistema Único de Saúde) and other healthcare systems, as it may provide perspectives for addressing a largely preventable problem [4].

Importantly, anemia, often driven by inadequate iron supplementation, is the most common health problem during pregnancy. It is a risk factor for maternal complications such as increased susceptibility to infections, increased risk of peripartum transfusion, preeclampsia, and placental abruption and may ultimately result in maternal death [2, 5]. In addition, anemia during this stage poses significant risks to the fetus, including intrauterine growth restriction, low birth weight, neurological impairment, and stillbirth [5, 6].

Given these risks, the World Health Organization recommends daily supplementation of 30–60 mg of elemental iron throughout pregnancy, starting from the time of pregnancy until delivery, in regions where the prevalence of anemia is equal to or greater than 20%, as is the case in Brazil [3]. When properly implemented, this measure reduces the risk of maternal anemia by approximately 70% and the risk of iron deficiency by 57% in pregnant women [3].

The diagnostic thresholds for gestational anemia vary by trimester in recognition of the physiological plasma volume expansion that occurs during pregnancy. According to the World Health Organization and the Centers for Disease Control and Prevention, anemia in pregnancy is defined as hemoglobin < 11 g/dL (hematocrit < 33%) in the first and third trimesters, and as hemoglobin < 10.5 g/dL (hematocrit < 32%) in the second trimester. These trimester-specific thresholds are relevant both for clinical diagnosis and for interpreting the epidemiological burden of anemia across gestation, since the second-trimester reference accommodates the hemodilution that accompanies normal pregnancy-related plasma volume expansion.

Despite these WHO recommendations and the proven effectiveness of this intervention, inadequate iron supplementation continues to be a significant problem for women, particularly in less developed countries, indicating the existence of structural barriers in the access to and implementation of this treatment. In this context, analyzing the factors responsible for nonadherence to iron supplementation during pregnancy is relevant, especially those related to inadequate prenatal care. Among these factors are economic vulnerability, maternal age, ethnicity, maternal education level, marital status, parity, lack of continuous professional guidance, and personal history, including the presence of comorbidities and drug use [1].

The identification and mitigation of these barriers are fundamental for the effectiveness of public policies aimed at preventing and controlling gestational anemia, contributing to improved maternal and child health indicators and achieving global sustainable development goals [1].

Because adequate iron supplementation during pregnancy depends not only on the prescription and dispensing of the supplement but also on the regularity, quality, and continuity of prenatal follow-up, the effectiveness of this intervention is closely tied to the adequacy of prenatal care itself. Previous work at our institution demonstrated that women who received adequate prenatal care showed higher adherence to ferrous sulfate supplementation.¹⁰ For this reason, the present study jointly examines sociodemographic factors associated with (i) the adequacy of iron supplementation during pregnancy and (ii) the adequacy of prenatal care follow-up, since these two outcomes are conceptually and operationally interrelated and cannot be fully understood in isolation.

Although sociodemographic and healthcare determinants of adherence to iron supplementation and of prenatal care utilization have been described in international guidance documents and in multisite Brazilian surveys,¹,⁸ few contemporary studies have jointly characterized these two outcomes within a single cohort of women delivering in a Brazilian quaternary teaching hospital serving predominantly SUS users. By examining both outcomes in the same 2023–2024 sample, the present study aims to identify specific vulnerable subgroups — notably adolescents, women who self-report substance use, unemployed women, and women whose prenatal care occurred outside the exclusive SUS trajectory — whose care gaps could be the focus of targeted public-health interventions at the interface between primary care, obstetric services, and psychosocial support.

## Methods

This cross-sectional study was conducted between March 2023 and May 2024 at the Maternity Unit of the Irmandade da Santa Casa de Misericórdia de São Paulo, a quaternary teaching hospital and reference center for obstetric care within the Brazilian Unified Health System (SUS). The institution serves a clinically and socioeconomically heterogeneous population composed predominantly of public health system users.

Women aged 18 years or older who were evaluated within 24 h postpartum were eligible for inclusion. The exclusion criteria included fetal death, multiple pregnancies, and the presence of major fetal malformations. All participants signed an informed consent form before their inclusion in the study. The study was approved by the Research Ethics Committee (CAAE 68850223.0.0000.5479).

The dataset analyzed in the present study overlaps with that used by Bertotti et al.,⁸ who examined the impact of iron supplementation on maternal hemoglobin concentrations and neonatal outcomes in the same cohort. The present analysis addresses a distinct research question—namely, the sociodemographic, obstetric, and health-service factors associated with inadequate iron supplementation and with inadequate prenatal care—and no individual-level results (e.g., hemoglobin concentrations, neonatal outcomes) are reproduced from that prior report. This sample overlap is disclosed here to ensure transparency for readers and to support appropriate interpretation of the complementary findings from the two analyses.

The sample size calculation was performed considering an expected prevalence of inadequate iron supplementation of approximately 59% based on a population-based Brazilian cross-sectional study by Cesar et al. [7], a 95% confidence level, and a maximum absolute margin of error of 5% points. Applying the standard formula for a single proportion, $\mathrm n\;=\;\mathrm Z^2\;\times\;\mathrm p\;\times\;(1-\mathrm p)\;/\;\mathrm d^2\;=\;{(1.96)}^2\;\times\;0.59\;\times\;0.41\;/\;{(0.05)}^2$, yielded a required sample size of 372 participants.

Data were collected via a structured questionnaire administered during the immediate postpartum period. The instrument included sociodemographic information, personal and obstetric history, and data related to prenatal care and iron supplementation during pregnancy.

The sociodemographic variables analyzed included maternal age, self-reported skin color, marital status, educational level, employment status, family income, use of liquor or illicit drugs, continuous medication use, and the presence of underlying diseases. The obstetric and healthcare variables included parity, history of preterm birth or low birth weight, gestational complications, prenatal care attendance, timing of prenatal care initiation, number of consultations, type of prenatal care (exclusively through the SUS, private, or mixed), use of ferrous sulfate, initiation of supplementation, and interruption of supplementation during pregnancy. Type of prenatal care was originally recorded in four categories (exclusively through the SUS, exclusively private, mixed public–private, and prenatal care initiated outside Brazil). Because the number of participants in the private, mixed, and overseas categories was small and did not reach the minimum cell frequencies required for reliable chi-square and Fisher’s exact testing, these three subgroups were aggregated into a single “mixed or non-SUS” category for statistical analysis, while preserving the SUS-exclusive category as the reference group.

Use of licit or illicit drugs during pregnancy was assessed through a single self-reported item in the structured questionnaire: “Did you use licit or illicit drugs during pregnancy? Yes/No.” Responses were recorded as a dichotomous variable. The questionnaire did not differentiate between specific substances (e.g., tobacco, alcohol, cannabis, cocaine), nor did it capture frequency, quantity, timing within pregnancy, or route of administration. This item therefore captures any self-reported exposure to psychoactive or licit substances during pregnancy; the resulting category combines women with potentially very different substance use patterns, which precludes dose–response or substance-specific analyses.

The selection of variables was based on previous evidence from the literature indicating that social, behavioral, and healthcare determinants are associated with adherence, preventive practices during pregnancy, and maternal and child outcomes [8].

Iron supplementation was considered adequate when the pregnant woman reported daily and continuous use of ferrous sulfate throughout pregnancy according to the World Health Organization recommendations, corresponding to a prophylactic dose of 30–60 mg of elemental iron per day starting in the first trimester without interruption. Supplementation was classified as inadequate when the pregnant woman did not use the supplement, initiated its use late, or reported interruption at any time during pregnancy.

The participants were categorized into two groups: adequate supplementation and inadequate supplementation or absence of supplementation. Prenatal care was considered adequate when the pregnant woman attended at least six prenatal consultations during pregnancy, according to national guidelines. Adequacy was evaluated quantitatively; the timing of prenatal care initiation and the content of consultations were not considered. The participants were categorized into two groups for this variable: adequate prenatal care and insufficient prenatal care.

Categorical variables are described using absolute and relative frequencies. Comparisons between groups were performed via Pearson’s chi-square test when all expected cell counts were ≥ 5, and via Fisher’s exact test when one or more expected cell counts were < 5, following standard recommendations for sparse contingency tables. In addition to p-values, crude odds ratios (ORs) with 95% confidence intervals (CIs) were calculated from the corresponding 2 × 2 tables using the Woolf (log-transformation) method to provide measures of effect size alongside the significance tests. The associations between sociodemographic, clinical, and healthcare variables and study outcomes were explored via bivariate analyses. The significance level was set at 5% (*P* < 0.05). Statistical analyses were performed via IBM SPSS Statistics software version 25 [9].

Reporting of this study followed the Strengthening the Reporting of Observational Studies in Epidemiology (STROBE) statement for cross-sectional studies. Several procedures were adopted to mitigate potential sources of bias. Data were collected by trained interviewers using a standardized, structured questionnaire applied uniformly to all participants during the immediate postpartum period, in order to minimize interviewer-related variability and differential misclassification. To reduce potential social desirability bias, participants were interviewed in a private setting, informed that responses were confidential and used exclusively for research purposes, and reassured that answers would not affect the care provided to them or their newborns. Eligibility criteria were applied consecutively to all women admitted for delivery during the study period in an effort to limit selection bias. After application of the exclusion criteria, the final analytic sample (*n* = 381) had complete data on all 24 sociodemographic, obstetric, and outcome variables used in the analyses, with no missing observations recorded for any variable; no imputation was therefore required and all bivariate analyses are based on the full sample of 381 participants. Unadjusted group frequencies and p-values from bivariate tests are reported throughout the Results section. To identify independent factors associated with inadequate iron supplementation, a multivariable binary logistic regression was performed with adequacy of iron supplementation as the dependent variable. Variables with *p* < 0.20 in the bivariate analyses were eligible for entry into the regression (use of drugs, employment status, family income, prenatal care adequacy, use of other vitamins during pregnancy, marital status, and type of prenatal care). A forward stepwise (conditional) method was applied, with entry probability of 0.05 and removal probability of 0.10. Model fit was assessed by the Cox & Snell and Nagelkerke pseudo-R² statistics and by the overall percentage of cases correctly classified. Adjusted odds ratios (aOR) with 95% confidence intervals are reported for the variables retained in the final model. A parallel multivariable model for the second outcome (adequacy of prenatal care) was not performed in this version; the associations between sociodemographic variables and prenatal care adequacy reported in Table 2 should therefore be interpreted as unadjusted bivariate estimates.

## Results

On the basis of the inclusion criteria, 381 parturients were selected for the study. With respect to self-reported skin color, 187 participants (49.1%) identified as pardo/a (mixed-race), 105 (27.6%) as White, 84 (22.0%) as Black, and 5 (1.3%) as Asian, reflecting the demographic profile of SUS users in the city of São Paulo. Overall, 176 of 381 women (46.2%) were classified as having adequate iron supplementation during pregnancy, while 205 of 381 (53.8%) were classified as having inadequate supplementation. With regard to prenatal care adequacy, 324 of 381 women (85.0%) had adequate prenatal care (≥ 6 consultations during pregnancy), and 57 of 381 (15.0%) had insufficient prenatal care. Comparative analyses of adequate versus inadequate supplementation and of adequate versus insufficient prenatal care were conducted on the basis of sociodemographic variables. The relationships that demonstrate statistical relevance are presented in Tables 1 and 2.

**Table 1:** Sociodemographic characteristics of the participants according to adequacy of gestational supplementation

Sociodemographic Characteristic	Group A: Adequate Supplementation (*N*, %)	Group B: Inadequate Supplementation (*N*, %)	Crude OR (95% CI)	Adjusted OR (95%CI)	*P* value
*Type of prenatal care*					0.073
* Public Health System*	168 (47.5%)	186 (52.5%)	1.00 (ref)	Not Retained	-
* Mixed or non-Public Health System*	8 (29.6%)	19 (70.4%)	2.15 (0.92–5.03)	Not Retained	-
*Drug Use*					0.001/0.005
* Yes*	15 (25.4%)	44 (74.6%)	2.93 (1.57–5.48)	2.54 (1.33–4.87)	-
* No*	161 (50.0%)	161 (50.0%)	1.00 (ref)	1.00 (ref)	-
Use of other vitamins during pregnancy					< 0.001/< 0.001
* Yes*	6 (12.5%)	42 (87.5%)	7.30 (3.01–17.6)	6.00 (2.45–14.72)	-
* No*	170 (51.1%)	163 (48.9%)	1.00 (ref)	1.00 (ref)	-
*Adequate prenatal care (≥ 6 consultations)*					0.001/0.033
* Yes*	161 (49.7%)	163 (50.3%)	0.36 (0.19–0.67)	0.47 (0.25–0.94)	-
* No*	15 (26.3%)	42 (73.7%)	1.00 (ref)	1.00 (ref)	-

**Table 2:** Sociodemographic characteristics of the participants according to adequacy of prenatal care

Sociodemographic Characteristic	Group C: Adequate Prenatal Care (*N*, %)	Group D: Insufficient Prenatal Care (*N*, %)	*P* value/Crude OR (95% CI)
*Age Group*			< 0.001
* 15–19 years*	22 (61.1%)	14 (38.9%)	5.05 (2.18–11.69)
* 20–24 years*	73 (83.9%)	14 (16.1%)	1.52 (0.71–3.26)
* 25–29 years*	94 (88.7%)	12 (11.3%)	1.01 (0.46–2.22)
* 30 + years*	135 (88.8%)	17 (11.2%)	1.00 (ref)
*Employment*			0.044
* Yes*	194 (88.2%)	26 (11.8%)	1.00 (ref)
* No*	130 (80.7%)	31 (19.3%)	1.78 (1.01–3.14)
*Type of prenatal care*			< 0.001
* Public Health System*	308 (87.0%)	46 (13.0%)	1.00 (ref)
* Mixed or non-Public Health System*	16 (59.3%)	11 (40.7%)	4.60 (2.01–10.53)
*Drug Use*			0.040
* Yes*	45 (76.3%)	14 (23.7%)	2.02 (1.02–3.99)
* No*	279 (86.6%)	43 (13.4%)	1.00 (ref)

With the exception of the type of prenatal care, drug use, employment status, and age group, the remaining sociodemographic variables and background characteristics analyzed did not show statistically significant associations with adherence to ferrous sulfate supplementation.

In Table 1, women who reported any use of licit or illicit drugs during pregnancy had nearly threefold higher odds of inadequate iron supplementation than those who did not (crude OR 2.93, 95% CI 1.57–5.48; *p* = 0.001). Women receiving prenatal care exclusively outside the SUS (mixed or non-SUS) had approximately twofold higher odds of inadequate supplementation than those receiving exclusively SUS-based care, although the confidence interval crossed unity (crude OR 2.15, 95% CI 0.92–5.03; *p* = 0.073), indicating an exploratory trend rather than a confirmed association. In Table 2, adolescents aged 15–19 years had a fivefold higher odds of insufficient prenatal care compared with women aged 30 years or older (crude OR 5.05, 95% CI 2.18–11.69), whereas women aged 20–24 (crude OR 1.52, 95% CI 0.71–3.26) and 25–29 years (crude OR 1.01, 95% CI 0.46–2.22) did not differ significantly from the reference category (overall *p* < 0.001 for the age trend). Unemployment was associated with higher odds of insufficient prenatal care (crude OR 1.78, 95% CI 1.01–3.14; *p* = 0.044). Women receiving non-SUS prenatal care had nearly fivefold higher odds of insufficient prenatal care compared with those receiving exclusively SUS-based care (crude OR 4.60, 95% CI 2.01–10.53; *p* < 0.001), and self-reported drug use was associated with twofold higher odds of insufficient prenatal care (crude OR 2.02, 95% CI 1.02–3.99; *p* = 0.040).

In the multivariable logistic regression with adequacy of iron supplementation as the outcome, three variables remained independently associated with the outcome after stepwise selection. Self-reported drug use during pregnancy was associated with a more than twofold higher adjusted odds of inadequate supplementation (aOR 2.54; 95% CI 1.33–4.87; *p* = 0.005). Non-use of other vitamins during pregnancy was the strongest independent correlate in the model (aOR 6.00; 95% CI 2.45–14.72; *p* < 0.001). Adequate prenatal care, defined as attending at least six prenatal consultations, was a protective factor (aOR 0.49; 95% CI 0.25–0.94; *p* = 0.033), meaning that women with adequate prenatal care had approximately half the odds of inadequate iron supplementation compared with those with insufficient prenatal care. Employment status, family income, marital status and type of prenatal care (SUS-exclusive vs. mixed/non-SUS) were eligible for entry into the model but did not remain in the final equation after stepwise selection (all *p* > 0.10), suggesting that the bivariate associations observed for these variables were partially explained by the behavioural and care-related factors retained in the model. The final model classified 61.7% of cases correctly; Cox & Snell R² = 0.106 and Nagelkerke R² = 0.142.

A direct cross-tabulation of the two outcomes was also performed. Of the 324 women with adequate prenatal care, 161 (49.7%) had adequate iron supplementation, compared with 15 of 57 (26.3%) among women with insufficient prenatal care (Pearson’s chi-square test; crude OR 2.77, 95% CI 1.48–5.18; *p* = 0.001). This pattern is consistent with the association between prenatal care attendance and iron supplementation reported in our cohort by Bertotti et al.¹⁰ and supports the interpretation that adequacy of supplementation is closely tied to the continuity and quality of prenatal follow-up.

Self-reported skin color was also examined in relation to both outcomes, after collapsing the four original categories into two (non-Parda [White or Asian] vs. Parda/Preta [mixed-race or Black]) to ensure adequate cell frequencies. No statistically significant association was observed between skin color and adequacy of iron supplementation (Parda/Preta vs. non-Parda: 44.6% vs. 50.0% adequate; crude OR 1.24, 95% CI 0.80–1.93; *p* = 0.342) or between skin color and adequacy of prenatal care (84.5% vs. 86.4% adequate; crude OR 1.16, 95% CI 0.61–2.19; *p* = 0.644). These null bivariate findings should be interpreted in light of the relative socioeconomic homogeneity of the sample (predominantly SUS users) and the limited statistical power for detecting moderate effects in this stratum, and are discussed further in the Limitations section.

## Discussion

The results of this study contribute to the understanding of how social and behavioral factors influence the effectiveness of iron prophylaxis during pregnancy. A previous study conducted at the same hospital concluded that women who received adequate prenatal care had increased adherence to ferrous sulfate supplementation during pregnancy [10]. Therefore, the present study also sought to investigate the relationships between sociodemographic variables and the adequacy of prenatal care. Inadequate adherence to supplementation represents an important public health issue and remains a challenge for prenatal care in Brazil, particularly among vulnerable populations.

Among the analyzed variables, self-reported drug use during pregnancy was significantly associated with inadequate adherence to ferrous sulfate supplementation in bivariate analysis (*p* = 0.001; crude OR 2.93, 95% CI 1.57–5.48) and remained independently associated after multivariable adjustment (aOR 2.54; 95% CI 1.33–4.87; *p* = 0.005). This finding reinforces the role of social and behavioural determinants in the effectiveness of preventive measures for maternal health and indicates that the association is not merely a reflection of other correlated sociodemographic factors.

The multivariable model also identified non-use of other vitamins during pregnancy as the strongest independent correlate of inadequate iron supplementation (aOR 6.00; 95% CI 2.45–14.72). This association likely reflects a shared underlying pattern of adherence to prenatal recommendations rather than a distinct causal pathway, since taking iron and other vitamins are parallel behaviours that depend on similar attitudes toward prophylactic care, access to consultations, and motivation. From a public health perspective, this finding suggests that interventions targeting general adherence to prenatal recommendations may have broader effects on iron supplementation specifically, and that women who decline or interrupt other vitamins during pregnancy may benefit from intensified counselling on iron prophylaxis. The protective effect of adequate prenatal care on supplementation adequacy also persisted after multivariable adjustment (aOR 0.49; 95% CI 0.25–0.94; *p* = 0.033), confirming that the continuity and frequency of prenatal follow-up are independent determinants of iron supplementation adequacy in this population. Notably, employment status, family income, marital status, and type of prenatal care (SUS-exclusive vs. mixed/non-SUS) lost statistical significance after adjustment, suggesting that the bivariate associations observed for these sociodemographic variables were partially mediated by the behavioural and care-related factors retained in the final model.

The association between psychoactive substance use and inadequate supplementation can be understood in the broader context of social vulnerability. Drug use during pregnancy is frequently associated with socioeconomic instability, social stigma, and weakened connections with healthcare services, which compromise both the continuity of prenatal care and adherence to regular medication use. Therefore, low adherence to iron supplementation in this group should not be interpreted solely as an individual failure of the pregnant woman but rather as a reflection of structural barriers in healthcare access and organization. Leal et al. highlighted that social vulnerability and stigma limit access to and the quality of prenatal care [11]. Furthermore, substance dependence during pregnancy has been associated with impaired self-care and reduced adherence to medical recommendations, as well as lower engagement with prenatal services, partly due to stigma and social vulnerability [12].

Although no statistically significant association was observed between the type of prenatal care and the adequacy of iron supplementation, a trend toward greater adherence was observed among women followed exclusively within the Brazilian Unified Health System than among those whose prenatal care occurred outside the country, within the private healthcare system, or through mixed public–private care (*p* = 0.073). Because this difference did not reach statistical significance and because the non-SUS subgroups (private, mixed public–private, and prenatal care initiated outside Brazil) were aggregated into a single comparison group for analysis — as detailed in the Methods section — this pattern should be interpreted as exploratory and hypothesis-generating. We therefore refrain from attributing the observed trend to specific causal mechanisms operating within any individual subgroup, since such mechanisms (for example, structural barriers faced by immigrant women or discontinuities associated with private insurance) were not directly measured in this study. As broader contextual information from the literature, Tomasi et al. [13] evaluated the quality of prenatal care within the primary care services of the SUS and demonstrated that the public healthcare network provides more consistent medication distribution and regular prenatal consultations, a finding that is consistent with — though not directly tested by — the descriptive pattern observed in our sample.

With respect to prenatal care adequacy, significant associations were observed between maternal age, employment status, type of healthcare service, and drug use. Corroborating previous findings, advanced maternal age has been associated with greater utilization and intensity of prenatal care, including a higher number of consultations and closer clinical monitoring. Studies have demonstrated that women aged 35 years and older are more likely to receive increased prenatal surveillance and are less likely to have inadequate prenatal care compared to younger women [8, 13–15]. On the other hand, drug use and unemployment have emerged as important barriers to adequate prenatal follow-up, reinforcing the influence of social inequalities on prenatal care organizations [16].

Another factor that may contribute to adherence to appropriate ferrous sulfate supplementation during pregnancy is clear and adequate counseling regarding potential adverse effects and the importance of medication use for both the mother and fetus. Additional factors that may contribute to nonadherence to iron supplementation, even when the medication is provided free of charge by the public health system, include individual perceptions and experiences, which were not directly evaluated in the present study. Among these are the adverse effects commonly associated with ferrous sulfate, such as nausea, epigastric pain, constipation, and alterations in bowel habits, which may lead pregnant women to spontaneously discontinue treatment. Previous studies have shown that pregnant women who receive health education are significantly more likely to adhere to iron supplementation, with odds ratios as high as 2.6 [17]. Furthermore, nutritional education interventions have been associated with improved compliance and reduced risk of anemia during pregnancy. These findings suggest that adequate counseling and patient understanding play a central role in treatment adherence [18].

Furthermore, data collection during the immediate postpartum period may be subject to recall bias because participants were required to remember the behaviors and practices that occurred throughout pregnancy. Social desirability bias must also be considered, as some women may have reported greater adherence to supplementation because of fear of judgment or because they believed this to be the socially expected response in a hospital setting. These aspects reinforce the understanding that medication adherence during pregnancy is a multifactorial phenomenon involving not only medication availability but also the woman’s experience with treatment, communication with healthcare professionals, and perception of risks and benefits. An additional limitation concerns the aggregation of comparison groups for the variable “type of prenatal care.” Women receiving exclusively private care, mixed public–private care, and prenatal care initiated outside Brazil were combined into a single “mixed or non-SUS” category because the number of participants in each of these subgroups was insufficient to meet the minimum expected cell frequencies required for reliable chi-square and Fisher’s exact testing. These three populations face substantively different structural barriers — including, respectively, out-of-pocket costs and insurance instability, fragmented transitions between systems, and challenges faced by immigrant women such as discontinuity of care and differences in healthcare protocols — and their aggregation inevitably obscures meaningful heterogeneity. As a consequence, any interpretation referring specifically to immigrant women or to the precariousness of private insurance should be regarded as exploratory and hypothesis-generating rather than confirmatory. Larger multicenter studies with sufficient representation of each subgroup will be required to disentangle the independent contribution of each prenatal care trajectory to adherence.

A further important limitation concerns the operational definition of prenatal care adequacy, which in the present study was based exclusively on the quantitative criterion of ≥ 6 prenatal consultations during pregnancy, following the Brazilian Ministry of Health guideline for low-risk pregnancies. This is an acknowledged weak proxy for overall prenatal care adequacy: it does not capture the timing of prenatal care initiation, the content or quality of each consultation (e.g., laboratory work-up, ultrasound monitoring, counseling on supplementation and warning signs), adherence to risk-stratified recommendations, or the individualized schedule required for women with high-risk pregnancies, who may have attended six or more consultations and still have received suboptimal care relative to their clinical needs. Conversely, a woman with a low-risk pregnancy who received fewer than six consultations but had each consultation performed at the recommended intervals with adequate content may have been misclassified as having insufficient prenatal care. This misclassification is non-differential with respect to the exposures examined and is therefore expected to bias associations toward the null; nonetheless, our estimates of the association between sociodemographic variables and prenatal care adequacy should be interpreted in light of this limited operational definition. Future studies should adopt composite indices of prenatal care adequacy (such as the Adequacy of Prenatal Care Utilization Index or the Kotelchuck index) that jointly consider timing of initiation, number of visits relative to gestational age, and indicators of process quality.

A further limitation concerns the assessment of the primary exposure and outcome. Adequacy of iron supplementation in this study was based exclusively on self-reported intake, without biochemical confirmation of iron status by serum ferritin, which was not available in the dataset. Hemoglobin was measured as part of routine peripartum care and is available in the same cohort; a parallel analysis of this dataset by Bertotti et al. demonstrated that women classified as having adequate supplementation had significantly higher mean hemoglobin (12.39 vs. 12.05 g/dL, *p* = 0.013) and a lower prevalence of maternal anemia (10.8% vs. 17.6%, *p* = 0.04) than those with inadequate supplementation, supporting the clinical relevance and construct validity of the adequacy classification used here.⁸ Nonetheless, the absence of ferritin precludes a direct assessment of iron stores, and future studies should incorporate biochemical markers alongside self-reported adherence.

Although a multivariable logistic regression was performed to identify independent determinants of inadequate iron supplementation, residual confounding from unmeasured variables cannot be excluded. In particular, the strong adjusted association observed for non-use of other vitamins (aOR 6.00) likely reflects a shared behavioural pattern of adherence to prenatal recommendations rather than an independent causal effect, and should be interpreted as a marker of overall adherence rather than as a distinct mechanism. The relatively modest pseudo-R² of the final model (Nagelkerke R² = 0.14) further indicates that a substantial proportion of the variance in supplementation adequacy is explained by factors not captured by the variables collected in this study, suggesting that future research should incorporate measures of health literacy, perceived risk, patient–provider communication and access barriers. In addition, the analysis of the second outcome — adequacy of prenatal care — was restricted to bivariate associations, as a parallel multivariable model was not performed in this version of the study; associations involving prenatal care adequacy in Table 2 should therefore be interpreted as unadjusted estimates.

Notably, other variables frequently associated with adherence to supplementation in the literature did not show significant associations in this study. This finding may reflect the relative socioeconomic homogeneity of the sample, which predominantly consisted of SUS users, thereby reducing the discriminatory power of these variables. Previous studies have identified multiple sociodemographic and structural determinants associated with inadequate prenatal care. Multiparity and unplanned pregnancy have been consistently associated with delayed initiation and lower adequacy of prenatal care, possibly reflecting reduced perceived risk and lower engagement with healthcare services. In addition, the absence of a partner has been associated with delayed initiation of prenatal care, highlighting the importance of social support in facilitating access to maternal health services. Furthermore, structural barriers such as distance to healthcare facilities, transportation availability, and related costs have been widely recognized as key determinants of inadequate prenatal care utilization. These findings reinforce that adherence to prenatal care is influenced by a complex interplay of individual, social, and systemic factors [8, 14, 19, 20].

Overall, the findings suggest that sociodemographic factors exert a greater influence on the adequacy of prenatal care than does direct adherence to iron supplementation. This finding indicates that inadequate supplementation may result from deficiencies in longitudinal follow-up, professional counseling, and overall quality of care rather than from individual characteristics. In this context, strategies aimed at strengthening the relationship between pregnant women and healthcare teams, combined with clear guidance regarding iron supplementation and proper management of adverse effects, may contribute to improved treatment adherence.

These patterns also invite a more explicit reflection on the structural and systemic barriers that shape iron supplementation adherence among pregnant women in Brazil, and on the intersecting axes of inequity that mediate exposure to such barriers. The association we observed between drug use and inadequate supplementation cannot be disentangled from the stigma that pregnant women who use psychoactive substances routinely encounter in healthcare settings, which may discourage disclosure, delay prenatal care initiation, and erode therapeutic relationships with providers. The organization of substance use care within the SUS, largely delivered through psychosocial care centers (CAPS and CAPS-AD) that operate in parallel to primary care and specialized obstetric services, tends to fragment the care pathway precisely for women whose clinical and social complexity would most benefit from integrated management; this fragmentation plausibly contributes to gaps in the prescription, dispensing, and monitoring of iron supplementation during pregnancy. It is important to emphasize, however, that such fragmentation is not what is prescribed by the guiding principles of Primary Health Care (Atenção Primária à Saúde, APS) in Brazil — on the contrary, care coordination is one of the foundational principles of the APS and of the SUS as a whole, and the organization of care is expected to be articulated through the primary care team as the ordenadora do cuidado. Similar fragmentation affects women who transition between public and private networks and those who initiate prenatal care abroad, for whom clinical records, prescriptions, and follow-up schedules are not systematically shared across services. These structural features interact with intersectional dimensions of disadvantage: in our cohort, 271 participants (71.1%) self-identified as Black or mixed-race (pardo), which is consistent with national and local patterns whereby Black and mixed-race (pardo) women are overrepresented among SUS users, face higher rates of inadequate prenatal care, and experience documented racial inequities in the quality of obstetric care, the social vulnerabilities we captured through sociodemographic variables (age, employment, substance use, type of prenatal care) cannot be read as race-neutral.⁷;⁹ Although the present sample size precluded formal interaction or stratified analyses by self-reported skin color, future work should explicitly examine how race, socioeconomic position, and substance use jointly structure access to and continuity of preventive interventions such as iron prophylaxis, and should inform equity-oriented responses that go beyond individual-level adherence and address the systemic barriers embedded in how care is organized and delivered.

From a public health policy perspective, several practical recommendations can be drawn more directly from the associations observed in our data. First, because drug use during pregnancy was significantly associated with inadequate iron supplementation (*p* = 0.001; crude OR 2.93, 95% CI 1.57–5.48) and also with inadequate prenatal care follow-up (*p* = 0.040; crude OR 2.02, 95% CI 1.02–3.99), our findings support the routine, non-stigmatizing screening of psychoactive substance use during prenatal consultations and the establishment of dedicated care pathways for pregnant women who use substances, integrating obstetric, mental health, and psychosocial support. Second, since adolescents aged 15–19 years had markedly lower adequacy of prenatal care compared with older age groups (*p* < 0.001), our findings support adolescent-specific prenatal care strategies — including earlier entry into prenatal care, partnerships with school health programs, and youth-friendly counseling — targeted at improving both the number and quality of consultations in this group. Third, because unemployment was associated with inadequate prenatal care (*p* = 0.044), the integration of prenatal care teams with social protection services (such as social workers embedded in primary care units and linkage with municipal social assistance) is directly supported by our data as a strategy to mitigate the impact of socioeconomic vulnerability on prenatal follow-up. Fourth, because women whose prenatal care took place outside the exclusive SUS trajectory showed significantly lower adequacy of prenatal care (*p* < 0.001) and a non-significant trend toward lower supplementation adherence (*p* = 0.073), our findings support the development of mechanisms that ensure continuity of care and standardized documentation of prenatal information across the public and private networks, so that transitions between systems do not translate into gaps in supplementation prescription and monitoring.

Beyond these data-derived recommendations, the recommendations outlined below are informed primarily by the broader literature on maternal health and adherence rather than derived directly from the specific findings of the present study, which was descriptive and exploratory in design. With that caveat, these results reinforce the need for strategies that focus not only on the availability of supplements but also on strengthening health education initiatives and promoting adherence to treatment. Educational campaigns directed toward pregnant women conducted in primary healthcare units, maternity hospitals, and communication platforms improve awareness regarding the importance of iron supplementation and reduce negative perceptions associated with its use. In addition, structured interventions during prenatal care, such as active counseling about possible adverse effects and strategies to minimize them, monitoring adherence in subsequent consultations, and using accessible educational materials, may contribute to greater treatment continuity. The involvement of community health workers in active outreach to pregnant women and the reinforcement of guidance through home visits also represent promising strategies, particularly in the context of greater social vulnerability. Thus, public health policies integrating health education, strengthening primary care, and longitudinal monitoring of pregnant women may significantly increase adherence to supplementation and consequently reduce the prevalence of gestational anemia and its adverse outcomes.

## Conclusion

Considering the importance of iron supplementation during pregnancy, this study sought to identify sociodemographic factors associated with inadequate implementation of this prophylactic measure among parturients receiving care at the Maternity Unit of the Irmandade da Santa Casa de Misericórdia de São Paulo, a hospital integrated into the Brazilian Unified Health System (SUS). Gestational anemia remains an important public health issue, affecting up to one-third of pregnant women in developing countries, and represents a significant marker of socioeconomic conditions and health system development.

Drug use during pregnancy was independently associated with low adherence to iron supplementation, both in bivariate analysis (*p* = 0.001; crude OR 2.93, 95% CI 1.57–5.48) and after multivariable adjustment (aOR 2.54; 95% CI 1.33–4.87; *p* = 0.005). The multivariable model also identified non-use of other vitamins during pregnancy as the strongest independent correlate (aOR 6.00; 95% CI 2.45–14.72), likely reflecting a shared behavioural pattern of adherence to prenatal recommendations, and adequate prenatal care as a protective factor (aOR 0.49; 95% CI 0.25–0.94). In the broader literature, self-reported substance use during pregnancy has been linked to lower regularity in prenatal care follow-up, attributed to social vulnerability and to the psychiatric and social consequences of substance use, which may impair self-care and engagement with health services.

Although not statistically significant, greater adherence to prophylaxis was observed among women who received prenatal care exclusively through the SUS. This descriptive pattern did not reach statistical significance (*p* = 0.073) and should therefore be regarded as exploratory and hypothesis-generating. Because the non-SUS subgroups (private, mixed public–private, and overseas-initiated care) were aggregated for analysis due to insufficient cell frequencies, any subgroup-specific explanation — such as fragmented transitions between systems or discontinuities in prophylactic measures — is not directly supported by the present data and requires confirmation in larger studies with adequately powered subgroup comparisons.

Finally, older pregnant women showed greater adherence to prenatal care, likely reflecting that increased maternal age is generally associated with earlier initiation of prenatal follow-up and a greater number of consultations. In contrast, socioeconomic and behavioral vulnerabilities emerged as factors associated with deficiencies in prenatal care follow-up and adherence to prophylaxis.

Overall, the results indicated that adherence to iron supplementation is closely related to the quality of prenatal care, adequate professional counseling, and longitudinal follow-up of pregnant women. Strategies aimed at preventing gestational anemia should, therefore, prioritize strengthening primary healthcare, with emphasis on early identification of pregnant women in vulnerable situations, active follow-up of missed appointments, clear guidance on the use of ferrous sulphate, including the management of adverse effects, and ensuring the regular dispensing of supplements.

Furthermore, continuous training of healthcare teams, integration with psychosocial support services, and adoption of nonstigmatizing approaches in the care of pregnant women who use substances are essential measures to increase adherence to supplementation recommendations and promote improved maternal and fetal health outcomes. After multivariable adjustment, self-reported drug use, non-use of other vitamins during pregnancy and inadequate prenatal care emerged as the independent determinants of inadequate iron supplementation in this cohort, supporting integrated public health strategies that combine behavioural counselling, continuity of prenatal care, and promotion of comprehensive prophylactic adherence as the most directly evidence-based interventions for reducing gestational anemia in vulnerable populations within the SUS. Addressing gestational anemia, therefore, requires not only the prescription of supplements but also public health policies and clinical practices that consider the social determinants of health and ensure comprehensive, equitable, and high-quality prenatal care.

## Supplementary Information

Supplementary Material 1.

## Data Availability

The datasets used and/or analysed during the current study are available from the corresponding author on reasonable request.
