# Supplementary material for: Factors associated with inadequate iron supplementation among pregnant women in the Brazilian Unified Health System: a cross-sectional study
Source: BMC Pregnancy Childbirth. 2026 Jul 1;26:1035. doi: 10.1186/s12884-026-09437-y (PMC13591904; doi:10.1186/s12884-026-09437-y)
Supplement: Supplementary file 1 [file 12884_2026_9437_MOESM1_ESM.docx]

**Iron Supplementation During Pregnancy**

**Questionnaire to be answered by the parturient:**

**Maternal age (years):**
15–19
20–24
25–29
Over 30

**Marital status:**
Single
Married
Stable union
Divorced
Widowed

**Skin color:**
White
Mixed (Pardo)
Black
Asian

**Did you use licit or illicit drugs during pregnancy?**
Yes
No

**Did you use any continuous medication during pregnancy?**
Yes. Which? ____________
No

**Do you have any underlying disease?**
Yes. Which? ____________
No

**GDM / GH** *(Gestational Diabetes Mellitus / Gestational Hypertension)*

**Education (years):**
0–4
5–8
9–11
More than 12

**Did you have paid work in the 12 months prior to or during pregnancy?**
Yes
No

**Family income (terciles):**
Lower (<1 minimum wage)
Intermediate (<2 minimum wages)
Higher (>2 minimum wages)

**Parity:**
None
One to two
Three or more

**Previous low birth weight infant:**
Yes
No

**Previous preterm birth (less than 37 weeks of gestational age):**
Yes
No

**Were there complications during pregnancy?**
Yes, requiring hospital admission
Yes, but no hospital admission
No

**Did you attend 6 or more prenatal visits?**
Yes
No

**Start of prenatal care (trimester):**
First
Second
Third
Did not attend

**Type of prenatal care:**
Public (in Brazil)
Private (in Brazil)
Abroad
Started abroad and finished in Brazil
Did not attend

**Did you receive vitamin supplementation during prenatal care (in addition to ferrous sulfate)?**
Yes
No

**Did you receive ferrous sulfate supplementation during prenatal care?**
Yes. For how long? ____________
No

**When did you start ferrous sulfate supplementation? (trimester)**
First
Second
Third
Did not receive

**Did you interrupt ferrous sulfate supplementation for any reason?**
Yes, interrupted
Did not interrupt
Did not receive

**Data to be completed by the researcher:**

**Maternal hemoglobin at admission:** _______

**Newborn birth weight:** _______

**Gestational age at birth:** _______
